# Supplementary material for: Genomic Insights of First ermB-Positive ST338-SCCmecVT/CC59 Taiwan Clone of Community-Associated Methicillin-Resistant Staphylococcus aureus in Poland
Source: Int J Mol Sci. 2022 Aug 6;23(15):8755. doi: 10.3390/ijms23158755 (PMC9369149; doi:10.3390/ijms23158755)
Supplement: Supplementary file 1 [file ijms-23-08755-s001.zip › Supplementary_Material_Table_S2.pdf]

Table S2. Antibiotic target genes and other resistance-associated genes and/or their regulatory regions, which gene variant or mutant or absence correlate with the manifestation of the resistance phenotype.

Gene	Gene product/ function/ KEGG code	Class of antibiotic	Antibiotics	PubMed ID
alr	Alanine racemase (EC 5.1.1.1)	Cycloserine	D-cycloserine	19748470; 24303782
ddl	D-alanine--D-alanine ligase (EC 6.3.2.4)	Cycloserine	D-cycloserine	24303782; 24033232
***fol*P/*dfr*A**	Dihydropteroate synthase (EC 2.5.1.15)	Sulfonamides	sulfamethoxazole, dapsone, sulfacetamide	15673783
***fus*A**	Translation elongation factor G	Fusidic acid	fusidic acid	17980694
***gyr*A**	DNA gyrase subunit A (EC 5.99.1.3)	Quinolones	fluoroquinolones	9293187
***gyr*B**	DNA gyrase subunit B (EC 5.99.1.3)	Quinolones	ciprofloxacin, levofloxacin	21693461; 22279180; 9293187
***inh*A, *fab*I**	Enoyl-[acyl-carrier-protein] reductase [NADH] (EC 1.3.1.9)	Isoniazid, Ethionamide, Triclosan	isoniazid, ethionamide, triclosan	18193820; 10869170; 8284673
***iso*-tRNA**	Isoleucyl-tRNA synthetase (EC 6.1.1.5)	Mupirocin	mupirocin	7929087
***kas*A**	3-oxoacyl-[acyl-carrier-protein] synthase, KASII (EC 2.3.1.179)	Isoniazid, Triclosan	isoniazid, triclosan	10428945
***mur*A**	UDP-N-acetylglucosamine 1-carboxyvinyltransferase (EC 2.5.1.7)	Fosfomycin	fosfomycin	8994972
rho	Transcription termination factor Rho	Bicyclomycins	bicyclomycins	8466900
***rpo*B**	DNA-directed RNA polymerase β subunit (EC 2.7.7.6)	Ansamycins	rifampicin	3050121; 15047531; 16723576
***rpo*C**	DNA-directed RNA polymerase β' subunit (EC 2.7.7.6)	Ansamycins	rifampicin	16723576
***rps*J (S10p)**	SSU ribosomal protein S10p (S20e)	Tetracyclines, Glycylcyclines	tigecycline	26124155; 26989065
**EF-Tu**	Translation elongation factor EF-Tu	Tetracyclines	tetracycline, minocycline, doxycycline	26989065
**S12p**	SSU ribosomal protein S12p (S23e)	Aminoglycosides	streptomycin	7934937
***par*E**	DNA topoisomerase IV subunit B (EC 5.99.1.3)	Quinolones	fluoroquinolones	26190223

tca*A**<br>tca*B**<br>***tcaR***	Membrane protein TcaA, teicoplanin resistance-associated<br>Teicoplanin resistance transporter, TcaB family (TcaB)<br>Teicoplanin-resistance-associated HTH-type transcriptional regulator TcaR	Glycopeptides	teicoplanin	11042376;<br>11150669;<br>11042376; 15060048
mep*A**<br>mep*R**	Multi antimicrobial extrusion protein (Na(+)/drug antiporter), MATE family of MDR efflux pumps<br>Transcriptional regulator, MarR family	Tetracyclines, Glycylcyclines	tigecycline	29039719
***nor*A**	MFS-type transporter quinolone resistance protein NorA	Quinolones	ciprofloxacin, acriflavin, norfloxacin	8431010; 2173911
arl*R**<br>arl*S**	Putative response regulator ArlR<br>Two component system histidine kinase ArlS (EC 2.7.3.-)	β-lactams, Glycolipopeptides	oxacillin, oritavancin	30924407
***mgr*A**	Transcriptional regulator MgrA (regulator of autolysis)	β-lactams, Glycolipopeptides	oxacillin, oritavancin	19072553
***gid*B**	16S rRNA (guanine(527)-N(7))-methyltransferase (EC 2.1.1.170)	Aminoglycosides	streptomycin	17238915
***gdp*D**	Glycerophosphoryl diester phosphodiesterase (EC 3.1.4.46)	Lipopeptides	daptomycin	21899450
***mpr*F**	L-O-lysylphosphatidylglycerol synthase (EC 2.3.2.3)	Lipopeptides	daptomycin	19289517; 16723576
***cls*A**	Cardiolipin synthase, bacterial type ClsA	Lipopeptides	daptomycin	29082727
***pgs*A**	CDP-diacylglycerol--glycerol-3-phosphate 3-phosphatidyltransferase (EC 2.7.8.5)	Lipopeptides	daptomycin	22238576
lia*F**<br>lia*R**<br>***lia*S**	Membrane protein LiaF (VraT), specific inhibitor of LiaRS (VraRS) signaling pathway<br>Cell envelope stress response system LiaFSR, response regulator LiaR (VraR)<br>Cell envelope stress response system LiaFSR, sensor histidine kinase LiaS (VraS)	Lipopeptides	daptomycin	21899450; 26020679

Legend: KEGG - Kyoto Encyclopedia of Genes and Genomes
