# Supplementary material for: Genomic Insights of First ermB-Positive ST338-SCCmecVT/CC59 Taiwan Clone of Community-Associated Methicillin-Resistant Staphylococcus aureus in Poland
Source: Int J Mol Sci. 2022 Aug 6;23(15):8755. doi: 10.3390/ijms23158755 (PMC9369149; doi:10.3390/ijms23158755)
Supplement: Supplementary file 1 [file ijms-23-08755-s001.zip › Supplementary_Material_Table_S3.pdf]

Table S3. Virulence factor genes (VFG) detected in genome of analyzed MRSA SO574/12 strain.

Function as VF	Gene	Product of the gene/ function	Source database
Adherence	*clf*A	Clumping factor ClfA, fibrinogen-binding protein	VFG001289
	clfB	Clumping factor ClfB, fibrinogen-binding protein	VFG001290
	ebp	Elastin binding protein EbpS	VFG001278
	map	Extracellular adherence protein of broad specificity Eap/Map	VFG001799
Adherence, biofilm formation	*ica*A	Polysaccharide intercellular adhesin (PIA) biosynthesis N-glycosyltransferase IcaA	VFG001285
	*ica*B	Polysaccharide intercellular adhesin (PIA) biosynthesis deacetylase IcaB	VFG001287
	*ica*C	Polysaccharide intercellular adhesin (PIA) biosynthesis protein IcaC	VFG001288
	*ica*D	Polysaccharide intercellular adhesin (PIA) biosynthesis protein IcaD	VFG001286
	*ica*R	Biofilm operon *ica*ABCD HTH-type negative transcriptional regulator IcaR	VFG001284
Adherence, MSCRAMMs	*sdr*C	Adhesin SdrC	VFG001279
	*sdr*E	Adhesin SdrE, similar to bone sialoprotein-binding protein Bbp	VFG001281
Antiphagocytosis	*cap*8A	Capsular polysaccharide synthesis enzyme Cap5A/ Tyrosine-protein kinase transmembrane modulator EpsC	VFG001297
	*cap*8B	Tyrosine-protein kinase EpsD/ Capsular polysaccharide synthesis enzyme Cap5B	VFG001298
	*cap*8C	Capsular polysaccharide synthesis enzyme Cap5C; Manganese-dependent protein-tyrosine phosphatase	VFG001299
	*cap*8D	Probable polysaccharide biosynthesis protein EpsC	VFG001300
	*cap*8E	UDP-N-acetylglucosamine 4,6-dehydratase	VFG001301
	*cap*8F	Capsular polysaccharide synthesis enzyme Cap5F	VFG001302
	*cap*8G	UDP-N-acetyl-L-fucosamine synthase	VFG001303
	*cap*8H	Capsular polysaccharide synthesis enzyme Cap8H	VFG001304
	*cap*8I	Capsular polysaccharide synthesis enzyme Cap8I	VFG001305
	*cap*8J	Maltose O-acetyltransferase	VFG001306
	*cap*8K	O-antigen flippase Wzx	VFG001307

Function as VF	Gene	Product of the gene/ function	Source database
	*cap*8L	Capsular polysaccharide synthesis enzyme Cap5L	VFG001308
	*cap*8M	Capsular polysaccharide synthesis enzyme Cap8M	VFG001309
	*cap*8N	Capsular polysaccharide synthesis enzyme Cap8N	VFG001310
	*cap*8O	UDP-N-acetyl-D-mannosamine dehydrogenase	VFG001311
	*cap*8P	UDP-N-acetylglucosamine 2-epimerase	VFG001312
Exoenzyme, protease	*clp*X	ATP-dependent Clp protease ATP-binding subunit ClpX	15927253
Exoenzyme, protease, stress protein	*clp*P	ATP-dependent Clp protease proteolytic subunit ClpP	VFG000077
Exoenzyme, lipase	*geh*	Lipase precursor	VFG001316
	lip	Triacylglycerol lipase	VFG004773
Exoenzyme, protease, cysteine protease	*ssp*B	Staphopain B precursor	VFG001295
	*ssp*C	Staphostatin B	VFG001294
Exoenzyme, protease, serine protease	*ssp*A	Glutamyl endopeptidase precursor, serine proteinase SspA	VFG001296
Exoenzyme, protease, zinc metalloproteinase	*aur*	Zinc metalloproteinase aureolysin	VFG001314
Exoenzyme, spreading factor	*hys*A	Hyaluronate lyase precursor	VFG001315
Immune evasion	*ads*A	Virulence-associated cell-wall-anchored protein SasH, 5'-nucleotidase	VFG002420
	chp	Chemotaxis-inhibiting protein CHIPS, phage-associated	VFG002422
	sbi	IgG-binding protein SBI	VFG002421
	scn	Involved in expression of fibrinogen binding protein, phage-associated	VFG002423
Immune evasion, adherence	*spa*	Protein A, von Willebrand factor binding protein Spa	VFG001313
Iron uptake, heme uptake	*isd*A	Cell surface protein IsdA, transfers heme from hemoglobin to apo-IsdC	VFG001318
	*isd*B	Cell surface receptor IsdB for hemoglobin and hemoglobin-haptoglobin complexes	VFG001317
	*isd*C	NPQTN cell wall anchored protein IsdC	VFG001319
	*isd*D	Heme transporter IsdDEF, membrane component IsdD	VFG001320
	*isd*E	Heme transporter IsdDEF, lipoprotein IsdE	VFG001321
	*isd*F	Heme transporter IsdDEF, permease component IsdF	VFG001322
	*isd*G	Heme-degrading monooxygenase, staphylobilin-producing	VFG001324

Function as VF	Gene	Product of the gene/ function	Source database
	*srt*B	NPQTN specific sortase B	VFG001323
Secretion system, type VII secretion system	*esa*A	Putative secretion accessory protein EsaA/YueB, phage SPP1 receptor	VFG002406
	*esa*B	Putative secretion accessory protein EsaB/YukD	VFG002407
	*ess*A	Putative secretion system component EssA	VFG002404
	*ess*B	Putative secretion system component EssB/YukC	VFG002408
	*esx*A	6 kDa early secretory antigenic target ESAT-6 (EsxA)	VFG002405
Toxin, membrane-acting, superantigen	*hlb*	Sphingomyelinase	VFG001798
	seb	Exotoxin, phage-associated superantigen enterotoxin SEB	VFG001802
	*sel*k	Superantigen enterotoxin SEK	VFG001327
	*sel*q	Exotoxin, phage-associated	VFG001326
Toxin, membrane-damaging, pore-forming, channel-forming involving alpha-helix-containing toxin	*hld*	RNAIII (delta-hemolysin)	VFG001292
Toxin, membrane-damaging, pore-forming, channel-forming involving β-sheet-containing toxin	*hly*/ *hla*	Cytolytic pore-forming protein, alpha-hemolysin	VFG001293
Toxin, membrane-damaging, pore-forming, channel-forming involving β-sheet-containing toxin (β-barrel), bicomponent leukocidin	*hlg*A	Cytolytic pore-forming protein S component, gamma-hemolysin HlgA	VFG001273
	*hlg*B	Cytolytic pore-forming protein F component, gamma-hemolysin HlgB	VFG001275
	*hlg*C	Cytolytic pore-forming protein S component, gamma-hemolysin HlgC	VFG001274
	*luk*F-PV	Panton-Valentine leukocidin chain F precursor	VFG001276
	*luk*S-PV	Panton-Valentine leukocidin chain S precursor	VFG001277

Legend: VF – virulence factor; VFG – virulence factor gene in virulence factor database, VFDB; MSCRAMM – microbial surface components recognizing adhesive matrix molecules
